# Supplementary material for: Intracellular Sugar Transporters Facilitate Cellulase Synthesis in Trichoderma reesei Using Lactose
Source: Biomolecules. 2023 Feb 4;13(2):295. doi: 10.3390/biom13020295 (PMC9953249; doi:10.3390/biom13020295)
Supplement: Supplementary file 1 [file biomolecules-13-00295-s001.zip › biomolecules-2039631-supplementary.pdf]

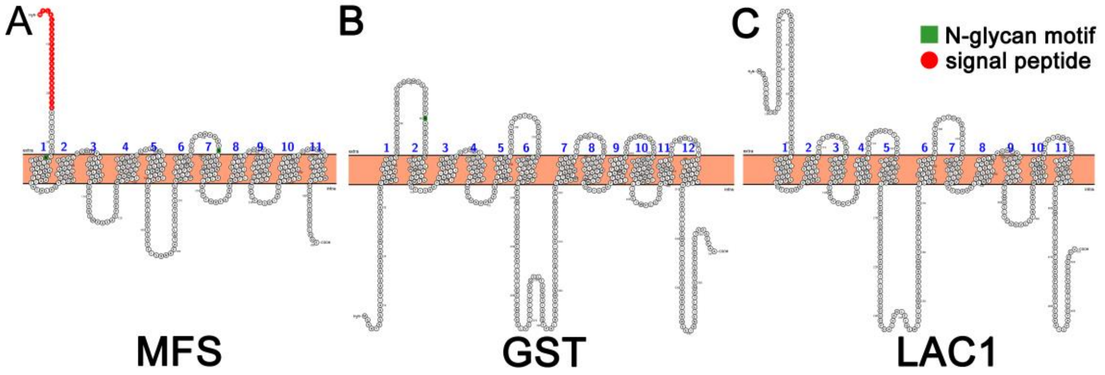

Figure S1 Bioinformatic analysis of transmembrane domains of sugar transporters MFS, GST, and LAC1 obtained from the webserver Protter (http://wlab.ethz.ch/protter/#). The green square represents N-glycosylation motif and red circle represents the signal peptide.

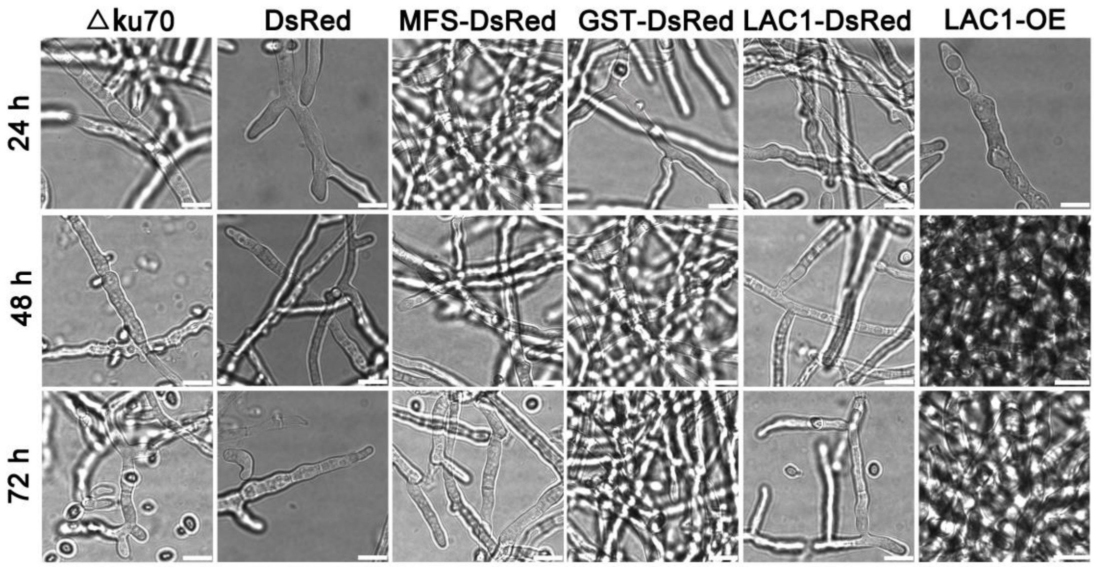

Figure S2 Brightfield images of T. reesei strains Δku70, DsRed, MFS-DsRed, GST-DsRed, LAC1-DsRed and LAC1-DsRed-OE (LAC1-OE) cultured in TMM+2% lactose at 24 h, 48 h, and 72 h, respectively. Scale bar = 10 μm.

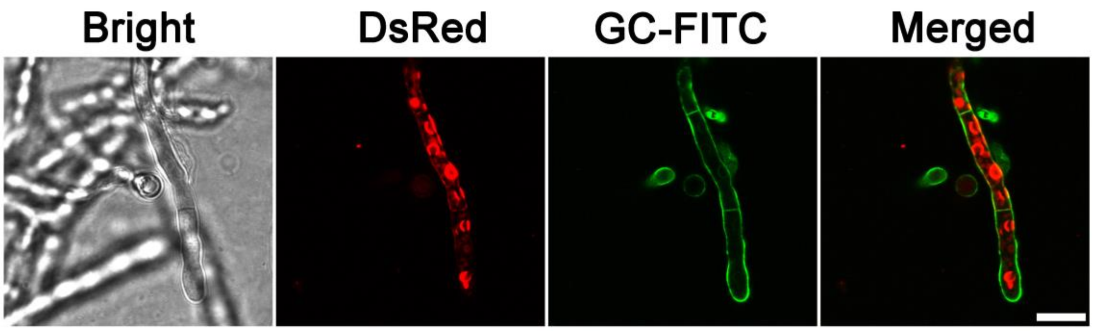

Figure S3 Confocal images of strain MFS-DsRed stained with GC-PEG-cholesterol-FITC, a green fluorescence dye for cell walls. Strain MFS-DsRed was grown on lactose for 72 h. Scale bar = 10 μm.

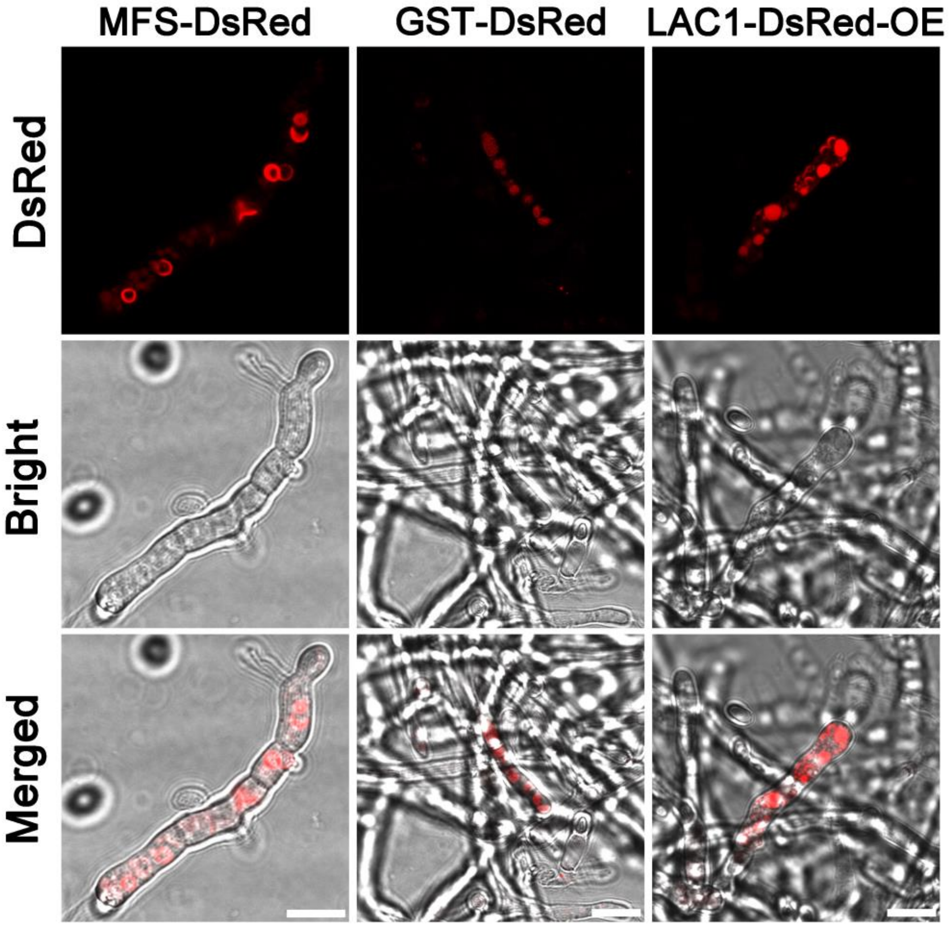

Figure S4 Cellular location of MFS-DsRed, GST-DsRed, and LAC1-DsRed-OE at the apical regions of recombinant strains MFS-DsRed, GST-DsRed, and LAC1-DsRed-OE cultured on lactose for 72 h. Scale bar = 10μm.

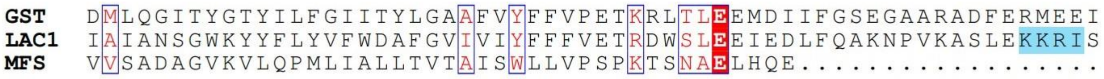

Figure S5 Bioinformatic analysis of ER localization signal motif of sugar transporters MFS, GST, and LAC1. The ER localization signal motifs KKXX were highlighted with blue background. The amino acids in red background and white character are strictly identical, in red character are similar in protein sequence, and in blue frame are similar across protein sequences.

Table S1 Primers used for gene amplification, verification, and qRT-PCR

Primer name	Sequence (5' → 3')
**Primers for qRT-PCR**	
q-crt1-F	GACTACTCGTGGCGACTCAT
q-crt1-R	GGACTCGGGCAGGAACAT
q-mfs-F	CCGGAGTCAACGATGGAG
q-mfs-R	CTGGCGGAGGTGGGTATT
q-gst-F	TGGCAAGCAGAGTGAAGC
q-gst-R	GCGGTTGTGGTGGATGAG
q-lac1-F	TTCGGCATCACCACTCAG
q-lac1-R	CAAGGACCAGCGACAAAA
q-sar1-F	TGGATCGTCAACTGGTTCTACGA
q-sar1-R	GCATGTGTAGCAACGTGGTCTTT
q-cel3a-F	ATGCGTTACCGAACAGCAGCTGC
q-cel3a-R	TGCGGCCTTCGCCTTGTCGTAC
q-cel7a-F	GCGGATCCTCTTTCTCAGAC
q-cel7a-R	TTGGCGTAGTAATCATCCCA
q-cel7b-F	ACTACACGGAGGAGCTCGACGACTT
q-cel7b-R	AAGGCATTGCGAGTAGTAGTCGTTG
q-bxl1-F	CGAGTTTGGCAGTGGTCTCT
q-bxl1-R	TGTGCGAACAAACAGCATGG
q-xyr1-F	TACCAAGTGCGATGGCTTAC
q-xyr1-R	CTCTCTCGGACATATTCGCA
q-ace3-F	CCCAAGTACTCGTGGCACAT
q-ace3-R	ATGGTGATGGGCCGATTGTT
q-clr2-F	CTCCCTGCTCAACATGGTCTTT
q-clr2-R	GGATGTGCTCTATGGCTTGGTT
q-xpp1-F	AGTTTGACGAGGATGCCGAC
q-xpp1-R	CGCTGTCGTCGAGAAAGTCTT
q-clr3-F	GACAGCACCGTCGATACTCACA
q-clr3-R	AGGTAGCAGACCAGCGAAAGGA
q-crt1-F1	CCTTTTCCAGCTTTGCCACC
q-crt1-R1	CTCTTCCAAAGTGCGTCCCT
q-stp1-F	GCAGTCCATCGGCATCCATA
q-stp1-R	TACCACCGAACGAGGCAAAG
q-xyl1-F	GCTCAAGCTCAACAGCGGATAT
q-xyl1-R	AGTCACAGGCACCGTCAAACAG
**Primers for gene amplification and verification in gene deletion**	
mfs-UP-F	ATTATTATGGAGAAACTCGAGAGCGGGCTTGAAGCTGTAG
mfs-UP-R	CCGTCACCAGCCCTGCTCGAGTCGCTTTGTCGAGGCTACG
mfs-DO-F	GTGAGGGTTAATTGCGCGGATCCGTGGTGACTGATACGCGAT

mfs-DO-R	CAGGTCGACTCTAGAGAGGATCCGCCATCTTGCCGCTTTTAC
gst-UP-F	ATTATTATGGAGAAACTCGAGGAGACGGTGTTACATGCTT
gst-UP-R	CCGTCACCAGCCCTGCTCGAGTTGTGAGGAGGAGAGACAGATA
gst-DO-F	GTGAGGGTTAATTGCGCGGATCCAATAGCGGAAGCACCGGGGC
gst-DO-R	CAGGTCGACTCTAGAGAGGATCCTGGGGCAACCTTCCACGCAA
lac1-UP-F	ATTATTATGGAGAAACTCGAGGCCAATGTCAAGGCGGTTT
lac1-UP-R	CCGTCACCAGCCCTGCTCGAGTGCGAGACGCTGTCATGCA
lac1-DO-F	GTGAGGGTTAATTGCGCGGATCCACAAGTGAAGGCGAGTGTAGTC
lac1-DO-R	CAGGTCGACTCTAGAGAGGATCCCTTTCCTGTCGCAAGAGCAA
Del-yz-UP-R	TCATTGACTGGAGCGAGGCGATGA
Del-yz-DO-F	GGGGATCAGCAATCGCGCATAT
Del-mfs-yz-UP-F	CTTCCGACTTGACGGCACC
Del-mfs-yz-DO-R	TTGCTACGGGGTCTGGGGAACT
Del-gst-yz-UP-F	ATATTGCCGCAACTTGGAGGACTG
Del-gst-yz-DO-R	CGGAGAACCGCACCCAACCTAT
Del-lac1-yz-UP-F	TTGGCCACCTGCATCTGTTGAAGC
Del-lac1-yz-DO-R	CACGGTTGCGTTGTGCTCATCA

Primers for gene expression at its endogenous locus and for gene over-expression

mfs-Red-UP-F	ATTATTATGGAGAAACTCGAGATGTACTTGAAGCTCATCAGCGC
mfs-Red-UP-R	CCGAGCCACCGCCACCCTCGAGCTCTTGATGCAGCTCGGC
mfs-Red-DO-F	GTGAGGGTTAATTGCGCGGATCCGTGGTGACTGATACGCGA
mfs-Red-DO-R	CAGGTCGACTCTAGAGAGGATCCCATAGCCCTGCCATCTTG
gst-Red-UP-F	ATTATTATGGAGAAACTCGAGATGGGCGCGCACACCGA
gst-Red-UP-R	CCGAGCCACCGCCACCCTCGAGAGCGGCCTTCTCAGCTTCAAC
gst-Red-DO-F	GTGAGGGTTAATTGCGCGGATCCAATAGCGGAAGCACCGGGGCG
gst-Red-DO-R	CAGGTCGACTCTAGAGAGGATCCCCGCAGCACCGGCGTAGTATCG
lac1-Red-UP-F	ATTATTATGGAGAAACTCGAGATGCAAGCCGCGCTGCTAC
lac1-Red-UP-R	CCGAGCCACCGCCACCCTCGAGAACATCGTCCCTACCATCTGGC
lac1-Red-DO-F	GTGAGGGTTAATTGCGCGGATCCACAAGTGAAGGCGAGTGTAGTC

lac1-Red-DO-R	CAGGTCGACTCTAGAGAGGATCCGGCAAAAAGCAGTATGGGAC
lac1-OE-UP	ACCCAATAGTCAATCTAGAATGCAAGCCGCGCTGCTAC
lac1-OE-DO	CTGCTGCTGCCGCTTCTAGAAACATCGTCCCTACCATCTGGC
Red-yz-UP-R	TCTCGAACTCGTGGCCGT
Red-yz-DO-F	GGGGATCAGCAATCGCGCATAT
mfs-R-yz-UP-F	CCTCCCTTCGGACTCTTT
mfs-R-yz-DO-R	TCATCACCTCCTCCACCCAACA
gst-R-yz-UP-F	GCCATTGATGCTGGTCCG
gst-R-yz-DO-R	CGGAGAACCGCACCCAACCTAT
lac1-R-yz-UP-F	GCGCTGTCCTGCAAACTG
lac1-R-yz-DO-R	GCTCACTTTCTGGCGGCGTTTT
